# The complete chloroplast genome sequence of *Lychnis kiusiana* (Caryophyllaceae)

**DOI:** 10.1080/23802359.2019.1639556

**Published:** 2019-07-16

**Authors:** Jiwon Yoon, Gi Heum Nam, Byoung Yoon Lee, Myounghai Kwak

**Affiliations:** National Institute of Biological Resources, Incheon, Republic of Korea

**Keywords:** chloroplast genome, *Lychnis kiusiana*, Caryophyllaceae, Silene, endangered species

## Abstract

The complete chloroplast genome sequence of *Lychnis kiusiana* Makino (Caryophyllaceae) was determined. The genome was 151,831 bp long, consisting of a large single-copy region (83,875 bp) and a small single-copy region (17,591 bp) separated by two inverted repeats (25,331 bp). The plastome contained 124 genes; 82 encode proteins, 34 encode tRNA, and eight encode rRNA. The gene quantity and order resembled those of a typical Caryophyllaceae plastome. Phylogenetic analysis using 79 gene sequences from 14 previously reported genomes of Caryophyllaceae showed that *Lychnis* clades were nested within the *Silene* genus, suggesting that *Lychnis* is a lower taxonomic rank than genus.

*Lychnis kiusiana* Makino is an endangered perennial Caryophyllaceae plant native to Korea and Japan. This species is distinguished from other *Lychnis* species by its feather-like, bright red petals, and its linear to narrowly lanceolate leaf blades. Recently, Japan and Korea have recognized that this species population is decreasing (Yamasaki et al. 2013). We aimed to investigate the genetic characteristics of the *L. kiusiana* chloroplast genome as initial basic research on the species to support a future conservation effort. Because this species has been designated recently as an endangered species by Korean government, detailed location information is not described here.

*Lychnis kiusiana* plant materials were cllected from its natural habitat in Pusan, Korea. A voucher specimen was deposited in the herbarium (KB) at National Institute of Biological Resources (NIBRVP0000628640). Plant genomic DNA was extracted from silica-gel dried leaf samples using an Exgene Plant SV kit (GeneAll, Korea). High-throughput sequencing was performed using the Illumina HiSeq 2500 platform (Illumina Inc., San Diego, CA, USA). The chloroplast genome was assembled *de novo* using a CLC_assembler v. 4.010.83648 (CLCbio, Aarhus, Denmark). Assembly errors and gaps were manually corrected through paired-end whole-genome sequencing read mapping using the CLC_mapper v. 4.010.83648 (CLCbio). Structural features and genes in the chloroplast genome were predicted using Ge-seq and Inverted Repeats Finder (IRF). The complete plastome sequence was submitted to the NCBI database under the accession number MG599084.

The complete chloroplast genome of *L. kiusiana* was circular and 151,831 bp in length. It consisted of a large single-copy region of 83,875 bp, a small single-copy region of 17,591 bp, and a pair of inverted repeats (IRa and IRb) of 25,331 bp. The length of the *L. kiusiana* chloroplast genome was similar to those previously reported for Caryophyllaceae chloroplast genomes. The complete chloroplast genome *of L. kiusiana* contained 124 genes, of which eight were ribosomal RNA genes, 34 were transfer RNA genes, and 82 were predicted protein-coding genes. The average GC ratio of the *L. kiusiana* chloroplast genome was 36.8%.

For phylogenetic tree construction, 75 gene sequences from 14 previously reported taxa were aligned over 67,380 bp. Seven genes were duplicated in the IR region and thus were counted only once. A phylogenetic tree was constructed based on the maximum-likelihood value of −167,213.71 using MEGA v.6. On the phylogenetic tree, *L. kiusiana* was nested within the *Silene* genus, along with other two *Lychnis* species (Figure 1). Currently, recognition of the *Lychnis* genus is controversial. Whereas Greuter (1995) recognized four subgenera, including a *Lychnis* subgenus within *Silene*, Oxelman and Lidén (1995) divided *Lychnis* and *Silene* into two separate genera based on nuclear ribosomal internal transcribed spacers and chloroplast *rps16* intron sequences. However, recent phylogenetic analyses of Caryophyllaceae, as shown here, support the hypothesis that *Lychnis* is nested within *Silene*. These results are consistent with previous phylogenetic studies using internal transcribed spacers and five chloroplast genomes as well as whole-genome sequences, revealing that *Lychnis* is nested within *Silene* (Greenberg and Donoghue 2011; Kang et al. 2017).

**Figure 1.**
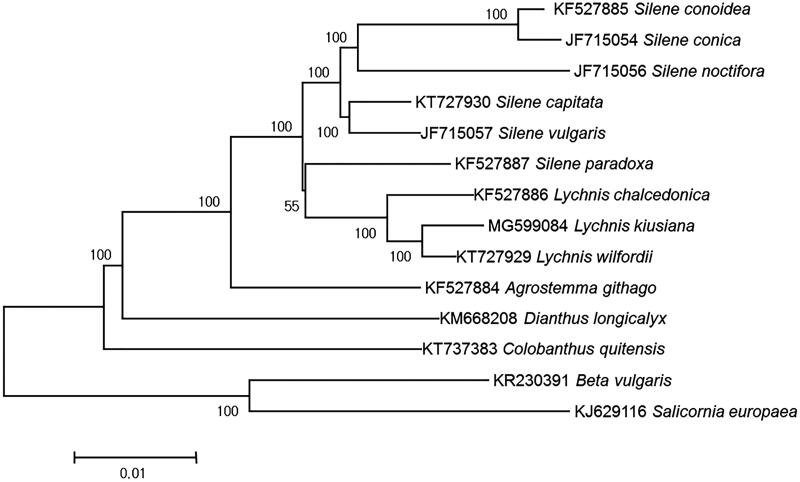
Phylogenetic tree of 14 taxa using maximum likelihood based on sequences of 75 protein-coding genes.
